# Smart Devices and Multimodal Systems for Mental Health Monitoring: From Theory to Application

**DOI:** 10.3390/bioengineering13020165

**Published:** 2026-01-29

**Authors:** Andreea Violeta Caragață, Mihaela Hnatiuc, Oana Geman, Simona Halunga, Adrian Tulbure, Catalin J. Iov

**Affiliations:** 1 Faculty of Mechanical, Industrial and Maritime Engineering, Ovidius University of Constanta, 900573 Constanta, Romania; andreea.caragata@365.univ-ovidius.ro; 2 Department Electronic and Telecommunication, Constanta Maritime University, 900663 Constanta, Romania; 3 Division of Data Science and Artificial Intelligence, Computer Science and Engineering, Chalmers University of Technology, 412 96 Gothenburg, Sweden; geman@chalmers.se; 4 Faculty of Electronics, Telecommunications and Information, Technology, National University for Science and Technology, POLITEHNICA Bucharest, 060042 Bucharest, Romania; simona.halunga@upb.ro; 5 Informatics and Electronics Department, “1 Decembrie 1918” University of Alba Iulia, 510009 Alba Iulia, Romania; 6 Department of Electronics, Telecommunications and Information Technology, “Gh. Asachi” Technical University of Iași, 700050 Iași, Romania; iovcatalin@etti.tuiasi.ro

**Keywords:** mental health, machine learning, biomedical signals, smart devices, remote sensing technology, heart rate variability, wearables, multimodal sensing, depressive disorder

## Abstract

Smart devices and multimodal biosignal systems, including electroencephalography (EEG/MEG), ECG-derived heart rate variability (HRV), and electromyography (EMG), increasingly supported by artificial intelligence (AI), are being explored to improve the assessment and longitudinal monitoring of mental health conditions. Despite rapid growth, the available evidence remains heterogeneous, and clinical translation is limited by variability in acquisition protocols, analytical pipelines, and validation quality. This systematic review synthesizes current applications, signal-processing approaches, and methodological limitations of biosignal-based smart systems for mental health monitoring. Methods: A PRISMA 2020-guided systematic review was conducted across PubMed/MEDLINE, Scopus, the Web of Science Core Collection, IEEE Xplore, and the ACM Digital Library for studies published between 2013 and 2026. Eligible records reported human applications of wearable/smart devices or multimodal biosignals (e.g., EEG/MEG, ECG/HRV, EMG, EDA/GSR, and sleep/activity) for the detection, monitoring, or management of mental health outcomes. The reviewed literature after predefined inclusion/exclusion criteria clustered into six themes: depression detection and monitoring (37%), stress/anxiety management (18%), post-traumatic stress disorder (PTSD)/trauma (5%), technological innovations for monitoring (25%), brain-state-dependent stimulation/interventions (3%), and socioeconomic context (7%). Across modalities, common analytical pipelines included artifact suppression, feature extraction (time/frequency/nonlinear indices such as entropy and complexity), and machine learning/deep learning models (e.g., SVM, random forests, CNNs, and transformers) for classification or prediction. However, 67% of studies involved sample sizes below 100 participants, limited ecological validity, and lacked external validation; heterogeneity in protocols and outcomes constrained comparability. Conclusions: Overall, multimodal systems demonstrate strong potential to augment conventional mental health assessment, particularly via wearable cardiac metrics and passive sensing approaches, but current evidence is dominated by proof-of-concept studies. Future work should prioritize standardized reporting, rigorous validation in diverse real-world cohorts, transparent model evaluations, and ethics-by-design principles (privacy, fairness, and clinical governance) to support translation into practice.

## 1. Introduction

This paper explores the core principles guiding the use of smart devices in mental health care, including how user engagement, privacy considerations, and the role of digital interactions can foster a therapeutic alliance. The conducted research suggests that when thoughtfully designed, digital tools can support the therapeutic relationship by enhancing communication and providing continuous insight into patients’ experiences [1,2], though concerns remain about potential impacts on the clinician–patient relationship [3].

As technological development accelerates, numerous sectors are undergoing significant transformations, and mental health monitoring is no exception. This introduction outlines the context in which smart devices and multimodal systems have increasingly become central instruments of mental health management, providing a framework for the main themes and questions addressed in the following chapters. In recent years, the integration of intelligent devices into healthcare has reshaped the way emotional well-being is observed and assessed, offering innovative and efficient methods for real-time tracking of psychological states. To fully understand these developments, it is essential to consider the theoretical foundations that support them. This paper explores the principles guiding the use of smart devices in mental health care, including user engagement, privacy considerations, and the ways in which digital interactions can foster a therapeutic alliance. Moreover, multimodal systems capable of collecting diverse types of data, such as physiological indicators, behavioral signals, and self-reported information, have the potential to enhance the effectiveness of mental health interventions. This section examines how these heterogeneous data streams converge to create a more holistic understanding of an individual’s mental state, thereby facilitating increasingly personalized care. Nevertheless, several challenges persist. Issues regarding data accuracy, user acceptance, and the ethical implications of digital technologies remain significant barriers. This paper addresses these challenges in detail, offering a critical analysis of how current tools may be refined and more effectively integrated into mental health practice.

This review seeks to integrate current research and outline potential directions for future studies into the use of smart systems in mental health care. Through a systematic examination of the existing literature, the following chapters aim to develop a coherent understanding of how smart devices and multimodal technologies may transform the monitoring and treatment of mental health conditions, highlighting both their potential and the challenges that accompany their implementation.

**Figure 1:**
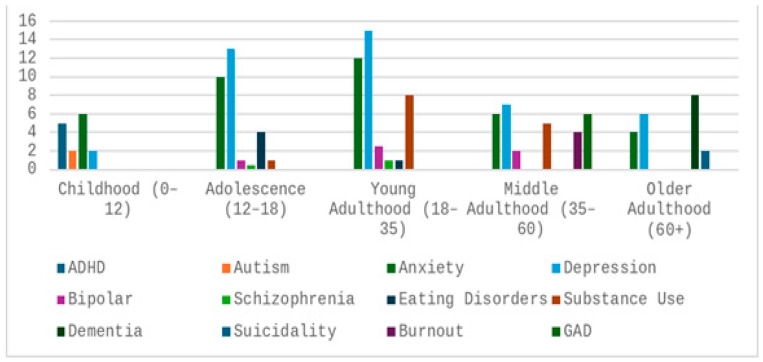
Distribution of major mental health disorders across age groups (%) [4].

Given the substantial variability of mental health disorders across different stages of life, it is essential to examine how these conditions manifest at various ages. Figure 1 provides an overview of major psychiatric disorders distributed by age group, illustrating, for instance, that conditions such as attention-deficit/hyperactivity disorder (ADHD) and autism typically emerge during early childhood [4]. As individuals transition into adolescence, the prevalence of depression, anxiety, and eating disorders increases. In young adulthood, mood and psychotic disorders reach peak incidence, while later life is marked by a growing prevalence of dementia-related conditions [5].

Several systematic reviews have examined aspects of digital mental health technologies. Mohr et al. (2017) [6] provided an early framework for personal sensing in mental health, focusing primarily on methodological considerations. Torous et al. (2019) [7] reviewed the broader landscape of digital psychiatry applications, while they specifically examined associations between mobile/wearable data and depressive symptoms. However, the authors have typically focused on either specific mental health conditions (primarily depression), single modalities (e.g., only smartphone data), or have not explicitly examined the integration of psychological theory with multimodal sensor data. Furthermore, most existing reviews predate recent advances in machine learning and do not comprehensively address the gap between research validation and real-world clinical implementation.

The present systematic review extends this literature in some important ways: (1) explicitly examining how psychological frameworks are integrated with multimodal sensing data across diverse mental health conditions; (2) synthesizing evidence across EEG, ECG, EMG, and environmental sensors in a unified framework; (3) critically evaluating the translational pathway from laboratory validation to clinical practice; and (4) identifying key technical, ethical, and practical barriers that must be addressed for successful implementation of smart mental health monitoring systems.

In response to the rapid proliferation of smart devices and multimodal sensing technologies in mental health care, this review addresses three primary research questions: (RQ1) the integration of psychological frameworks with physiological, behavioral, and contextual data; (RQ2) the evidence regarding the validity and reliability of multimodal sensing in detecting conditions such as stress, anxiety, and depression; and (RQ3) the technical, ethical, and practical barriers limiting the translation of these technologies into routine clinical practice.

## 2. Materials and Methods

This review is conducted following the Preferred Reporting Items for Systematic Reviews and Meta-Analyses (PRISMA) 2020 guidelines (Supplementary Materials) to ensure transparency and reproducibility of the review process [8]. A comprehensive literature search is performed across multiple electronic databases to identify relevant studies on smart devices and multimodal systems for mental health monitoring. The PubMed/MEDLINE, IEEE Xplore Digital Library, Web of Science, Scopus, PsycINFO, and ACM Digital Library databases were searched from 2013 to 2026. The protocol for this review was not prospectively registered in a public repository. However, the review methodology was defined a priori before the start of the study, including the research questions, eligibility criteria, search strategy, and data extraction process. These methodological decisions were consistently followed throughout the review in accordance with PRISMA.

**Figure 2:**
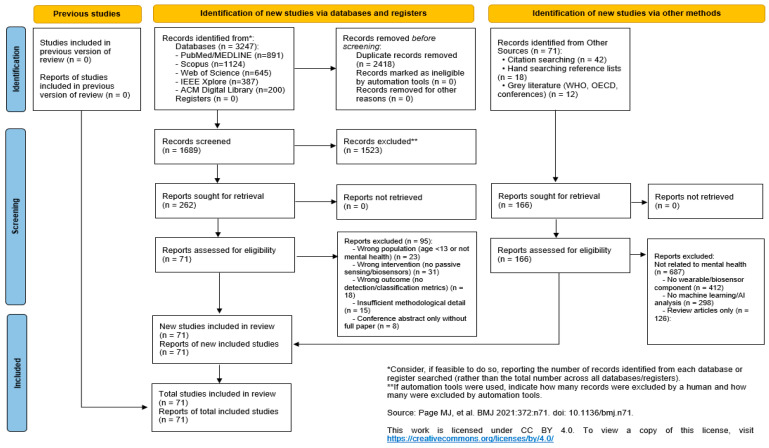
PRISMA 2020 flow diagram showing the study selection process [8].

The inclusion criteria for targeted original research articles included randomized controlled trials (RCTs) and cohort, cross-sectional, and pilot studies, published in English between 2013 and 2026 in peer-reviewed journals or conference proceedings. Selected studies focused on adult or mixed-age populations at risk of mental health conditions, evaluating the use of smart devices and multimodal sensing systems for monitoring mental states (such as depression, anxiety, or stress), physiological biomarkers (e.g., HRV, EEG patterns), or relevant clinical outcomes. Studies are excluded if they are systematic reviews, meta-analyses, editorials, commentaries, or protocols lacking results, as well as case reports. Research focused exclusively on populations with severe cognitive impairment or dementia is omitted to avoid confounding the interpretation of mental health signals. Furthermore, the review excluded studies relying solely on traditional clinical assessments without smart device integration or those using non-portable laboratory equipment. Methodologically, papers with a high risk of bias or insufficient detail for quality assessment are rejected, alongside any publications where the full text is not accessible in English or through institutional resources. Search strings combined four key domains: mental health, wearable/biosensing technology, physiological signals, and machine learning/artificial intelligence. After the database search, two investigators independently screened all records at each stage of the review process. Any disagreements were resolved through discussion, with fewer than 5% of cases requiring adjudication. Inter-rater reliability was high, with Cohen’s kappa values of κ = 0.85 for title and abstract screening and κ = 0.89 for full-text assessment, indicating a very strong level of agreement beyond chance. The final inclusion set comprised 71 studies, of which 42 provided sufficient quantitative data to be included in the meta-analysis (Figure 2). Risk of bias was evaluated narratively due to heterogeneity in design and metrics across studies. Reporting completeness, sample robustness, and validation strategies are examined.

Assessment of Study Characteristics:

1. Study characteristics by sensor type: EEG-based studies: *n* = 18; ECG/HRV-based studies: *n* = 32; EMG-based studies: *n* = 6; and multimodal sensing: *n* = 15;
2. Study characteristics by mental health condition: Depression/MDD: *n* = 41; anxiety disorders: *n* = 18; PTSD: *n* = 6; and multiple conditions: *n* = 6;
3. Study characteristics by ml method: Traditional ML (SVM, random forest, etc.): *n* = 28; deep learning (CNN, LSTM, and neural networks): *n* = 24; ensemble methods: *n* = 12; and Bayesian approaches: *n* = 7;
4. Publication year distribution: Between 2013 and 2016: *n* = 6; between 2017 and 2019: *n* = 14; between 2020 and 2022: *n* = 22; and between 2023 and 2026: *n* = 29.

The reviewed literature highlights five major research directions on the use of biosignals and multimodal systems in mental health monitoring. The largest theme is depression detection and monitoring, where studies mainly explore EEG signals (multiscale entropy, functional connectivity, and deep learning/transformers models, as well as home neurofeedback), ECG/HRV (classical and nonlinear indicators, with a clear evolution towards modern ML models), and, more recently, facial EMG as a biomarker of low affectivity. The second theme focuses on stress and anxiety management, including biofeedback interventions (EMG and combinations with virtual reality), meta-analyses supporting the relationship between HRV and anxiety, and ML applications for predicting stress levels.

Less represented, but clinically relevant, are studies on PTSD, where EEG neurofeedback and EMG biomarkers (startle) suggest promising, but still preliminary, results. In parallel, an important cross-cutting theme concerns technological innovations, from in-ear EEG electrodes and wearable magnetoencephalography (MEG) systems, Optically Pumped Magnetometers (OPM-MEG), to robust processing methods for artifact removal and integrated remote sensing technology/smartphone/smartwatch platforms for large-scale data collection. Finally, the literature also includes emerging intervention directions (real-time EEG-guided stimulation) and a set of papers that provide socioeconomic context (global burden, digitalization), justifying the need for accessible and scalable solutions.

The selection of papers is guided by strict inclusion criteria, focusing on original research, including randomized controlled trials (RCTs) and cohort, cross-sectional, and pilot studies, published in English between an initial search date of January 2013 and January 2026; last updated 15 January 2026.

Eligible studies targeted adult or mixed-age populations at risk of mental health conditions, specifically evaluating the use of smart devices and multimodal sensing systems for monitoring mental states, physiological biomarkers (e.g., HRV, EEG), or clinical outcomes. A thematic analysis of the included literature revealed six predominant cross-cutting themes: Depression detection and monitoring (37%); stress/anxiety management (18%); PTSD/trauma (5%); technological innovations for monitoring (25%); brain-state-dependent stimulation/interventions (3%); and socioeconomic context (7%). Descriptive Summarization: The review synthesizes findings descriptively, such as the following:

“Machine learning models achieved accuracy rates ranging from 78% to 94% in controlled settings” (i); “67% of studies had sample sizes <100 participants” (ii); “89% are proof-of-concept investigations” (iii); and “Only 23% of studies reporting validation in independent cohorts” (iv).

These are descriptive statistics about study characteristics, not pooled effect sizes from meta-analysis.

The review integrates findings across modalities (EEG, HRV, and EMG) and applications (depression, anxiety, and PTSD) through qualitative description and comparison, rather than statistical pooling.

Specific heterogeneity issues are identified as follows:

1. Diverse outcome measures: Different classification metrics (accuracy, sensitivity, specificity, and AUC, F1-score) (i); various feature extraction methods (ii); and inconsistent diagnostic criteria (iii).
2. Varied methodologies: Different sensor types and manufacturers (i); inconsistent acquisition protocols (ii); diverse preprocessing pipelines (iii); and multiple machine learning algorithms (iii).
3. Study design differences: RCTs, cohort studies, cross-sectional studies, pilot studies (i); laboratory vs. ambulatory settings (ii); varied sample sizes (most <100 participants) (iii); and different populations and demographics (iv).
4. Technical variability: EEG—Different electrode configurations, sampling rates, and frequency bands (i); HRV—Different recording durations, and time-domain vs. frequency-domain vs. nonlinear measures (ii); and EMG—Different muscle groups and electrode placements (iii).

Meta-analysis requires sufficient homogeneity to meaningfully pool results. When studies use fundamentally different methods, measures, and populations, statistical pooling produces invalid or misleading conclusions.

Complete Search Syntax with Boolean Operators

Primary Search String (Applied to all databases with syntax adaptations)

(“mental health” OR “depression” OR “anxiety” OR “psychological wellbeing” OR

“psychiatric disorder*” OR “mood disorder*” OR “major depressive disorder” OR “MDD” OR

“generalized anxiety disorder” OR “GAD” OR “PTSD” OR “post-traumatic stress”)

AND

(“wearable*” OR “biosensor*” OR “physiological sensing” OR “passive sensing” OR

“mobile sensing” OR “digital biomarker*” OR “sensor technology” OR “smart watch*” OR

“fitness tracker*” OR “activity tracker*”)

AND

(“EEG” OR “electroencephalogra*” OR “ECG” OR “electrocardiogra*” OR “EMG” OR

“electromyogra*” OR “heart rate variability” OR “HRV” OR “PPG” OR “photoplethysmogra*” OR

“galvanic skin response” OR “GSR” OR “electrodermal activity” OR “EDA”)

AND

(“machine learning” OR “artificial intelligence” OR “AI” OR “deep learning” OR

“neural network*” OR “classification” OR “prediction” OR “detection” OR “recognition” OR

“algorithm*” OR “predictive model*”)

Database-Specific Adaptations

PubMed/MEDLINE:

((“mental health”[MeSH Terms] OR “depression”[MeSH Terms] OR “anxiety disorders”[MeSH Terms]

OR “mood disorders”[MeSH Terms]) AND (“wearable electronic devices”[MeSH Terms] OR

“biosensing techniques”[MeSH Terms] OR “monitoring, physiologic”[MeSH Terms]) AND

(“electroencephalography”[MeSH Terms] OR “electrocardiography”[MeSH Terms] OR

“electromyography”[MeSH Terms] OR “heart rate”[MeSH Terms]) AND (“machine learning”[MeSH Terms]

OR “artificial intelligence”[MeSH Terms] OR “deep learning”[MeSH Terms]))

AND (2013:2026[pdat])

Web of Science:

TS = ((“mental health” OR depression OR anxiety OR “psychiatric disorder*”) AND

(wearable* OR biosensor* OR “physiological sensing” OR “digital biomarker*”) AND

(EEG OR “heart rate variability” OR HRV OR ECG OR EMG OR PPG) AND

(“machine learning” OR “artificial intelligence” OR “deep learning” OR classification))

AND PY = (2013–2026)

IEEE Xplore:

((“All Metadata”:”mental health” OR “All Metadata”:depression OR “All Metadata”:anxiety)

AND (“All Metadata”:wearable OR “All Metadata”:biosensor OR “All Metadata”:“physiological sensing”)

AND (“All Metadata”:EEG OR “All Metadata”:ECG OR “All Metadata”:EMG OR “All Metadata”:“heart rate variability”)

AND (“All Metadata”:“machine learning” OR “All Metadata”:“artificial intelligence” OR “All Metadata”:“deep learning”))

AND (2013:2026)

Scopus:

TITLE-ABS-KEY((“mental health” OR depression OR anxiety OR “mood disorder*”) AND

(wearable* OR biosensor* OR “passive sensing” OR “digital biomarker*”) AND

(eeg OR ecg OR emg OR “heart rate variability” OR hrv OR ppg) AND

(“machine learning” OR “artificial intelligence” OR “deep learning” OR algorithm*))

AND PUBYEAR > 2012 AND PUBYEAR < 2027

PsycINFO:

DE = (“Mental Health” OR “Major Depression” OR “Anxiety Disorders” OR “Mood Disorders”) AND

DE = (“Wearable Technology” OR “Biosensors” OR “Physiological Monitoring”) AND

DE = (“Electroencephalography” OR “Electrocardiography” OR “Heart Rate”) AND

DE = (“Machine Learning” OR “Artificial Intelligence” OR “Neural Networks”)

Limited to: 2013–2026

Additional Search Methods:

1. Citation Tracking: Forward and backward citation searching of included studies using the following: Scopus’ citation tracker (i); Web of Science’s Cited Reference Search (ii); and Google Scholar “Cited by” function (iii).
2. Hand Searching and Manual review of reference lists of the following: Included systematic reviews (i); key meta-analyses on digital mental health (ii); and recent review articles (2020–2026) (iii).
3. Gray Literature: WHO Digital Health Repository (i); OECD Health Policy Papers (ii); and conference proceedings such as IEEE EMBC, ACM CHI, and AAAI (iii).

Conditions for meta-analysis: Minimum of 5 studies with similar population characteristics (i); outcome measures (ii); and study design (iii).

Statistical Methods: Random-effects model (DerSimonian-Laird method) (i); heterogeneity assessed using I^2^ statistic (ii); publication bias: Funnel plots and Egger’s test (iii); sensitivity analyses: Leave-one-out method (iv); and subgroup analyses by sensor type and ML algorithm (v).

Software: R version 4.3.0 and Packages meta, metafor, and dmetar.

Data Extraction Method: Standardized data extraction form created in Microsoft Excel as a tool (i); pilot testing form piloted in 5 studies and refined (ii); and extraction protocol based on independent extraction by two reviewers; and weekly calibration meetings during the extraction phase and monthly progress reviews with the senior author.

Data Items Extracted Study Characteristics: First author, year of publication (i); study design (RCT, cohort, cross-sectional, or case–control) (ii); country of origin (iii); and funding source (iv).

Population Characteristics: Sample size (total, intervention/control) (i); age (mean ± SD, range) (ii); gender distribution (iii); mental health diagnosis (DSM-5/ICD-11 criteria) (iv); severity of condition (v); and comorbidities (vi).

Technology Details: Type of sensor(s) used (i); specific physiological signals measured (ii); duration of monitoring (iii); sampling frequency (iv); data collection setting (lab, ambulatory, or home-based) (v); and device specifications (make, model) (vi).

Machine Learning Methods: Algorithm type (SVM, random forest, CNN, LSTM, etc.) (i); feature extraction methods (ii); training/validation/test split (iii); cross-validation approach (iv); and preprocessing techniques (v).

Outcomes: Primary outcome measures (i); classification accuracy (%) (ii); sensitivity and specificity (iii); AUC-ROC values (iv); precision and recall (v); F1-score (vi); and confusion matrices (when reported) (v).

Bias and Quality: Risk of bias assessment scores (i); methodological limitations noted by authors (ii); and conflicts of interest declared (iii).

## 3. Overview of Smart Devices in Mental Health: Application and Evidence Synthesis

In recent years, digital technologies have begun to play an increasingly influential role in mental health care. The incorporation of tools such as wearable sensors, dedicated health applications, and network-enabled devices represents a notable shift in the way mental health conditions are monitored and addressed. At the same time, these devices can also enhance the therapeutic alliance by facilitating communication between sessions and offering clinicians richer, continuous insight into a client’s daily experiences and progress.

This chapter outlines several categories of smart technologies, grouped according to the mental health challenges they aim to track, while examining their functions, the design features that shape user engagement, the ethical implications surrounding data protection, the difficulties associated with integration into existing healthcare systems, and the broader impact on clinician–client interactions.

A wide range of smart devices now exists, each contributing differently to mental health support. Wearable devices, for instance, can gather information on physiological indicators such as sleep patterns or heart rate, whereas mobile applications allow individuals to document mood changes or engage in therapeutic exercises, including those inspired by cognitive-behavioral therapy. These technologies rely on advanced algorithms and real-time data analysis to tailor interventions to each user’s circumstances. The move toward digital solutions illustrates a broader transformation toward more participatory models of mental health care, encouraging individuals to take an active role through continuous monitoring and self-reflection.

User participation is important to the success of mental health applications. Existing research consistently demonstrates that the overall design of an application—including its interface, usability, and visual clarity—strongly influences whether individuals continue to use it and derive value from it. When an app is easy to navigate, aesthetically coherent, and intuitive, engagement tends to be higher. Personalized feedback, such as guidance on next steps or visualizations of progress, can further strengthen motivation and foster a sense of responsibility for one’s own well-being. The interplay between thoughtful design and sustained user involvement is therefore essential; poorly designed applications risk deterring users and diminishing therapeutic outcomes. As a result, prioritizing user-centered design principles is fundamental for the effective implementation of smart mental health technologies.

### 3.1. Wearable Dry-Electrode EEG Headsets for Depression Monitoring

In recent years, a variety of wearable Electroencephalogram (EEG) headsets—such as the Emotiv EPOC X and Muse S—have become commercially available, employing dry or semi-dry electrodes. These systems record brain activity and transmit data wirelessly, typically through Bluetooth, making them compatible with mobile platforms designed for at-home monitoring or neurofeedback training. Research published after 2021 demonstrates that measures such as frontal alpha asymmetry and theta power can be collected with reasonable reliability outside controlled laboratory environments. Moreover, early randomized clinical trials indicate that home-based neurofeedback may help reduce symptoms in individuals with depression or post-traumatic stress disorder [9,10]. Despite these promising developments, several limitations remain. Compared with laboratory-grade EEG equipment, wearable devices generally exhibit a lower signal-to-noise ratio and are more susceptible to artifacts. In addition, most are marketed primarily as wellness products and lack formal authorization for diagnostic or clinical use.

**Figure 3:**
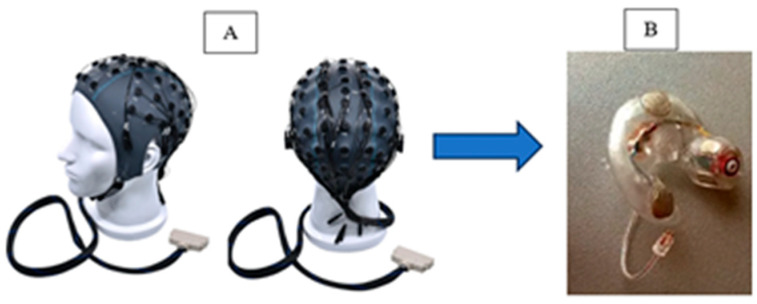
Comparison between conventional scalp EEG and novel in-ear EEG devices (adapted from [11]). (**A**) traditional scalp EEG setups, traditional wet-electrode EEG caps with conductive gel, (**B**) new, in-ear EEG devices.

Figure 3 shows how technology has advanced from traditional scalp EEG setups, traditional wet-electrode EEG caps with conductive gel, to new, in-ear EEG devices. These miniaturized sensors enable the monitoring of brain activity in a much more discreet and portable way, paving the way for continuous mental health tracking outside of lab environments.

Closed-loop TMS—EEG platforms: The combination of Transcranial Magnetic Simulation (TMS) coils with advanced EEG platforms has enabled stimulation to be delivered in alignment with specific phases of ongoing brain activity, such as the prefrontal alpha rhythm. Within this technological setup, the phase of the EEG signal is computed in real time, allowing TMS pulses to be administered at precisely targeted moments—typically at the peaks or troughs of alpha oscillations. This type of implementation relies on specialized research systems, including those provided by ANT Neuro and MagVenture. Findings from early pilot investigations [12,13] indicate that TMS delivered in synchrony with EEG activity produces a stronger modulation of prefrontal–cingulate networks compared to stimulation administered without phase targeting. Nonetheless, the approach remains largely experimental and entails substantial technical demands, ranging from the need for effective electromagnetic shielding and artifact reduction to the accurate management of stimulation latency.

EEG-guided neurofeedback software for PTSD commercial platforms, such as Brain Master Discovery and Neuro Guide, now offer clinically oriented EEG systems designed to support neurofeedback protocols. These tools deliver real-time visual or auditory feedback linked to a patient’s brain activity to encourage specific neural patterns—for example, enhance alpha activity or decrease elevated beta activity. Recent systematic reviews [14,15] report that standardized neurofeedback protocols can lead to moderate reductions in PTSD symptoms. Even so, the literature points to several important constraints, including substantial variability in the protocols employed and inconsistencies in the quality of the supporting evidence. Moreover, insurance coverage for these types of interventions remains limited.

### 3.2. Wearable MEG with OPM-MEG

New headset-based MEG systems have emerged that rely on scalp-mounted OPM sensors rather than conventional cryogenic SQUID technology. These sensors can detect femtotesla-level magnetic fields close to the scalp, and they are built into custom 3D-printed headsets and are typically paired with lightweight magnetic shielding setups. Recent research [16,17] has shown that OPM-MEG configurations are capable of reliably capturing auditory responses, such as the M50/M100 components and the 40 Hz steady-state response in both adults and children. Despite these advancements, several challenges persist: The systems still require magnetic shielding, remain costly, are limited to specialized research environments, and currently lack comprehensive normative datasets.

### 3.3. Concrete ECG and HRV Tools in Mental Health

#### 3.3.1. Wearable ECG Patches for Stress and Anxiety Monitoring

Single-electrode chest patches—such as VitalPatch, Zio, and BioPatch—enable continuous ECG monitoring for extended periods ranging from several days to multiple weeks. These devices track cardiac activity in real time by measuring the intervals between heartbeats and then upload the data to cloud platforms for storage and automated processing. After analysis, a wide array of heart rate variability (HRV) metrics can be derived, including time-domain indicators like RMSSD, frequency-domain ratios such as LF/HF, and more advanced nonlinear measures such as entropy. Recent research [18,19] consistently links reduced HRV with heightened anxiety and stress. In PTSD populations, these wearable sensors have also been used to identify hyperarousal episodes, with HRV decreases frequently occurring prior to symptom escalation. As a result, this technology holds promise for long-term stress monitoring in everyday environments, offering objective physiological indicators that complement self-reported measures and enabling timely, app-based interventions. Nonetheless, several challenges remain: Extended wear can cause discomfort, adherence varies across individuals, and HRV measures are highly sensitive to confounding influences such as caffeine intake, physical exertion, and sleep quality.

#### 3.3.2. HRV-Guided Biofeedback Apps for Depression and PTSD

Smartphone-based HRV biofeedback tools—such as HeartMath, Respira, and various clinically oriented prototypes paired with ECG sensors—offer a modern digital method for supporting emotional regulation and reducing stress. These systems typically guide users through paced breathing exercises while ECG or PPG sensors deliver real-time HRV feedback, often presented as coherence scores or through resonant-frequency breathing guidance. Randomized clinical trials published since 2020 [20,21] have reported meaningful reductions in depressive symptoms and PTSD-related distress following 4–8 weeks of HRV biofeedback practice, with improvements largely attributed to increases in vagally mediated HRV. Because the approach is low-cost, non-invasive, and easily integrated into digital mental health or telemedicine platforms, it holds considerable promise as an accessible therapeutic tool for depression, anxiety, and PTSD. Even so, its success depends heavily on consistent user engagement, and non-specific relaxation or placebo effects cannot be fully excluded. Additionally, standardized protocols for HRV biofeedback remain insufficiently developed.

#### 3.3.3. Consumer Wearables: ECG/Photo-Plethysmography (PPG) for Stress Tracking in Daily Life

Popular consumer wearables such as the Apple Watch (Series 6 and later), Fitbit Sense, and Oura Ring can estimate HRV using single-lead ECG recordings or PPG-based measurements. These devices compute the intervals between successive heartbeats and generate daily or night-time HRV metrics, with integrated algorithms identifying potential “stress” episodes and linking the data to digital health applications. Large real-world datasets [22] have shown that lower nocturnal HRV is associated with higher levels of depression and anxiety. Wearable devices have also been employed to monitor HRV longitudinally in individuals with affective disorders, enabling the prediction of relapse risk. As such, these technologies can deliver scalable insights into autonomic function, support early warning detection systems, and contribute to digital phenotyping efforts. Nevertheless, HRV derived from consumer wearables is vulnerable to measurement noise, relies on proprietary algorithms with limited transparency, and currently lacks clinical validation for formal decision-making.

### 3.4. Concrete EMG Tools in Mental Health

#### 3.4.1. EMG Biofeedback for Anxiety and Stress

Surface electromyography (sEMG) systems—typically positioned on the frontalis or trapezius muscles—are commonly paired with biofeedback platforms, such as Thought Technology’s ProComp or the NeXus system, to facilitate relaxation training. These sensors measure muscle activity in real time, while the accompanying software delivers visual or auditory cues that help users gradually reduce muscular tension. A 2021 meta-analysis [23] reported that EMG-based biofeedback leads to meaningful reductions in symptoms of generalized anxiety, with effect sizes comparable to those achieved through traditional relaxation methods. More recent research [24] has integrated EMG biofeedback with virtual reality environments, showing greater decreases in stress levels than virtual reality (VR) alone. Clinically, this approach is particularly valuable for individuals experiencing tension-related anxiety or stress linked to muscle contraction patterns, and it can be incorporated into cognitive-behavioral therapy (CBT) protocols. Nevertheless, the method requires multiple training sessions, placebo effects cannot be entirely ruled out, and access to the necessary equipment remains largely restricted to specialized treatment centers.

#### 3.4.2. EMG for Emotion Recognition in Affective Disorders

Surface electromyography (sEMG) electrodes placed on facial muscles—most commonly the zygomaticus major and corrugator—are used to capture subtle muscular activity linked to emotional expression. These sensors can identify micro-movements that arise during affective tasks, such as viewing emotional stimuli or engaging in social interactions. In research settings, facial sEMG is employed to evaluate reduced emotional responsiveness in depression or heightened reactivity in anxiety disorders. Recent work [25,26] has shown that sEMG activity patterns can distinguish depressed from non-depressed individuals, as well as anxious from relaxed states, with machine learning models achieving classification accuracies exceeding 70%. Clinically, this technology has the potential to offer objective indicators of emotional reactivity and to strengthen digital phenotyping strategies in psychiatry. Nonetheless, its use remains largely confined to laboratory environments because facial EMG requires controlled conditions and is highly vulnerable to movement artifacts, electrode misplacement, and environmental noise.

#### 3.4.3. EMG for PTSD and Trauma-Related Hyperarousal

**Figure 4:**
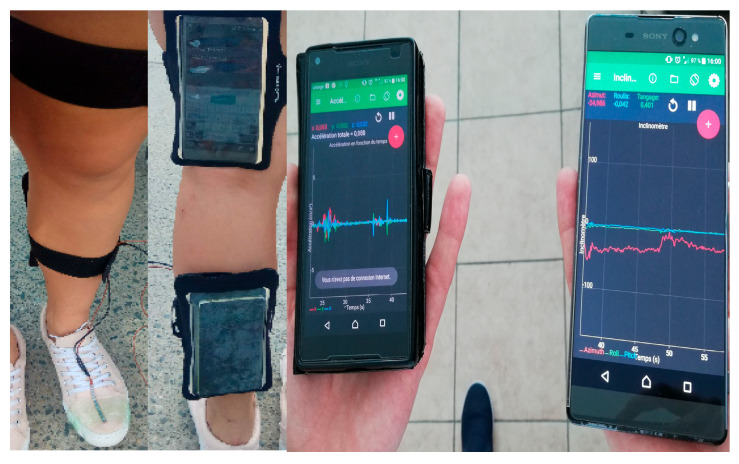
The system for gait recognition using a mobile phone and the Physics Toolbox interface (adapted from [27]).

Wearable electromyography (EMG) bands placed on the forearm or trapezius are increasingly used to measure startle reactions and muscle hyper-reactivity in individuals with PTSD. These sensors can be employed both in controlled startle-response paradigms and in everyday monitoring, and they are frequently paired with ECG or EDA devices to capture broader indicators of sympathetic activation. A set of studies published between 2020 and 2022 [27] showed that heightened EMG startle responses are a persistent feature of PTSD but can be attenuated through EMG-assisted biofeedback interventions. Early pilot research has also documented improvements in sleep and reductions in reactivity to trauma-related cues following such training. Clinically, this technology offers promise as an objective marker of hyperarousal, with potential applications in personalizing treatment plans and tracking therapeutic progress (Figure 4). Nevertheless, current protocols vary considerably, the ecological validity of laboratory-based tasks remains limited, and robust large-scale randomized trials are still lacking.

Figure 4 shows a prototype system for monitoring gait that uses common everyday devices, like smartphones equipped with inertial measurement units (IMUs), combined with resistive flex sensors. While it is initially created for tracking walking patterns and aiding in rehabilitation, this configuration demonstrates how remote sensing technology solutions can pick up on behavioral markers that are relevant to mental health, such as slow psychomotor responses in depression or changes in gait seen in schizophrenia [28].

### 3.5. Multimodal Systems for Monitoring Mental Health

A key strength of multimodal systems lies in their capacity to enhance the precision of mental health assessments. Conventional evaluation methods—typically based on interviews or self-report questionnaires—remain useful but are vulnerable to influences such as stigma, limited self-insight, or response biases. In contrast, multimodal approaches incorporate physiological data, including heart rate variability, galvanic skin response, and sleep metrics, offering objective indicators of psychological states. When these diverse data streams are combined, clinicians can form a more detailed and accurate picture of a patient’s mental health, ultimately supporting more informed therapeutic decisions. The use of advanced analytical methods, including artificial intelligence (AI), can further sharpen these evaluations by enabling predictive modeling that anticipates changes in mental state over time.

Physiological signals validated as markers of mental disorders within multimodal systems have become an especially valuable focus of current research. Recent findings show that biometric measures—such as fluctuations in heart rate variability—closely align with levels of stress and anxiety, making them critical elements in comprehensive mental health care frameworks.

For example, Sheikh et al. [29] reviewed recent advances in flexible smart sensors, where devices evolve from simple signal acquisition elements to systems capable of analysis and decision-making, through the integration of artificial intelligence. The authors present two complementary AI components: (1) machine learning algorithms (from classical to deep learning) used for preprocessing, pattern recognition, and multimodal fusion, and (2) artificial/neuromorphic synapses as hardware support for energy-efficient and biologically inspired processing. The novel functionalities obtained through this fusion (advanced data analysis, perception, and “smart” decisions) are synthesized, and representative applications such as human activity monitoring, artificial sensory systems, and soft/humanoid robotics are discussed. Epidermal multimodal systems combining ECG, GSR, and respiration with ML algorithms for mental fatigue classification (reported ~89% prediction rate in one example) and multimodal robotic platforms for object recognition (in one case, 100% accuracy is reported) are mentioned. The authors also emphasize that the full integration of sensors–ML–artificial synapses remains relatively limited in the current literature. Finally, practical challenges are highlighted: Updating models on devices in real time, robustness of synaptic components to mechanical deformations, interfacing between different materials, and the need for system-level co-design.

Observations like this underscore how emerging technologies can strengthen psychological interventions by enabling real-time feedback and continuous tracking capabilities that traditional assessment methods generally cannot provide.

A key component of these systems is the incorporation of validated physiological signals. EEG offers important insights into neural activity and cortical rhythms and is widely used to identify emotional or attentional disturbances. Surface EMG captures fine-grained muscle activity, from somatic tension linked to anxiety to subtle facial expressions associated with affective processing. Likewise, ECG and derived measures such as HRV serve as sensitive indicators of autonomic functioning and emotional regulation, with established relevance for monitoring stress, depression, and PTSD. When combined with other metrics—such as galvanic skin response or sleep-related data—these signals contribute to a comprehensive view of physiological reactivity and individual resilience. The following sections, therefore, focus on biosignal modalities commonly used in mental health monitoring—neural (EEG/MEG), cardiac (ECG/HRV), and neuromuscular (EMG)—and summarize the main processing pipelines, analytical methods, and evidence supporting their clinical relevance.

#### 3.5.1. Neural Signals: EEG and MEG

Electroencephalography (EEG) and MEG are fundamental tools in mental health research because they offer a direct window into brain activity. EEG is widely used because it is accessible and easy to carry around, making it accessible for many labs and clinical settings. On the other hand, MEG delivers much better spatial detail, but it needs specialized and expensive equipment, which is not always easy to set up. Both techniques play an important role in identifying biomarkers associated with conditions like depression, schizophrenia, bipolar disorder, and anxiety.

Preprocessing of EEG/MEG Signals

When working with EEG and MEG recordings, it is important to be aware that they can easily pick up unwanted noise or artifacts, necessitating rigorous preprocessing procedures. Typically, the process includes several key steps: first, applying a band-pass filter that targets frequencies between 0.5 and 50 Hz to focus on the signals most relevant to brain activity. Next, a notch filter at 50 or 60 Hz is used to eliminate interference from power lines. Independent Component Analysis (ICA) is then employed to separate genuine neural signals from artifacts caused by eye movements or muscle activity.

Finally, the data is often segmented into time-based segments or epochs, and baseline normalization is performed to make sure the data is comparable across different trials [30].

Spectral Analysis

The most traditional method involves frequency decomposition through the Fourier Transform (1):

(1) $$\hat{f}\left(\varepsilon \right)=\int_{-\infty }^{\infty }f\left(x\right)e^{-i2\pi \varepsilon x}dx,\forall \varepsilon \in R$$

Notes:

*f*(*x*)—the signal in the time (or space) domain.

$\hat{f}\left(\varepsilon \right)$—the Fourier transform of *f*.

Є—the frequency variable (Hz if *x* = *t*).

This allows us to measure the activity levels across the main EEG frequency bands: delta (0.5–4 Hz), theta (4–8 Hz), alpha (8–12 Hz), beta (12–30 Hz), and gamma (above 30 Hz).

- Depression is often associated with reduced alpha activity and increased beta power.
- Schizophrenia has been linked to disrupted gamma oscillations and reduced phase synchrony.
- Anxiety correlates with elevated beta activity and altered theta rhythms.

#### 3.5.2. Recent Methodological Advances

Lightweight multi-disorder EEG analysis [31]

Single-channel EEG recordings are analyzed using the Discrete Wavelet Transform (DWT), which enables the extraction of features such as approximate entropy, fuzzy entropy, permutation entropy, and spectral entropy. Multiple machine learning classifiers—including SVM, k-NN, Naive Bayes, and LDA—are trained on publicly available datasets related to schizophrenia, epilepsy, and depression. The findings are encouraging, with the models achieving strong performance; notably, depression is identified with an accuracy of 89.9%, a particularly striking result given that the analysis relied solely on data from a single EEG channel.

Temporal scale optimization in depression detection [32]

They applied multiscale entropy analysis across different time scales to better understand the data. By experimenting with classifiers, such as K-Nearest Neighbors (KNN), linear discriminant analysis (LDA), and support vector machines (SVM), they discovered that the best accuracy—reaching about 96.4%—occurred at an intermediate scale, specifically at scale 3.

Deep learning with EEG transformers for depression diagnosis [33]: The study proposes a method for predicting major depressive disorder (MDD) and depression severity using resting EEG spectral features and psychometric (questionnaire) data, separately and in combination. The authors evaluate both the binary classification (MDD vs. healthy) and regression for the Beck Depression Inventory (BDI) score.

Methods Sample: A total of 71 participants (42 healthy, 29 MDD), 18–59 years; clinical criteria and exclusion criteria are explained.

EEG: At rest, with eyes open/closed; recording on Brain Products’ 128-channel system, analysis on 96 channels; preprocessing with ICA (EEGLAB); extracted relative powers on bands (delta–gamma, 1–35 Hz).

Psychometrics: BDI + Big Five (IPIP), aggression, and emotional/social intelligence (EmIn).

Dimensionality reduction: PCA applied separately on the EEG and questionnaires.

Models: Classical methods (e.g., ridge/logistic regression, random forest, gradient boosting, MLP) and recurrent networks (SimpleRNN, LSTM), evaluated with nested cross-validation.

Results—Regression (BDI prediction): An LSTM on EEG data (especially on delta) obtains R^2^ = 0.742 and MAE = 6.114, reported as a major improvement over ridge regression.

EEG biomarkers (from ablation): Delta and alpha bands appear to be the most informative; beta/gamma have lower predictive utility (possibly also due to muscle artifact contamination).

Importance of psychometric predictors: Rumination (31.2%), age (27.9%), and hostility (18.5%) account for ~75% of the feature importance for severity.

Classification (MDD vs. control): The authors report very high performance for logistic regression, including ROC-AUC = 1.000 on spectral/combined EEG, plus high specificity and MCC values (in conclusions).

Semantic neural signatures in depression and suicidality [34]

Chen et al. (2024) [34] propose a methodological framework for robust construction of EEG functional networks in major depressive disorder (MDD), starting from resting-state recordings with eyes open (5 min), 19 channels, 256 Hz. The dataset includes 34 MDD and 30 controls, but in the actual analysis, 30 MDD and 28 controls are available. Preprocessing included 50 Hz notch filtering, 0.5–50 Hz band-pass, ICA artifact removal, re-referencing to average reference, and then segmentation of a stable interval (100 s after the first 60 s) into 10 s windows, resulting in 580 segments; the delta–gamma bands and the total band (0.5–50 Hz) are analyzed. The performances of the studies are evaluated by statistical tests and F-score, and the PLV + AT combination is reported to be the most reliable.

While EEG/MEG capture neural activity directly, cardiac biosignals offer a complementary view of autonomic regulation. In particular, ECG-derived HRV provides a practical and scalable marker of sympathetic–parasympathetic balance, making it well-suited for monitoring stress reactivity and emotion regulation in both laboratory and real-world settings.

Taken together, this body of work illustrates a clear and consistent advancement in the application of EEG-based methods for depression research (Table 1). In [35], Trenado et al. (2026) investigated the role of frontal EEG as a biomarker for predicting clinical response to intranasal ketamine/esketamine in major depression, in a retrospective study conducted in a real-life clinical setting (August 2021–June 2024). The analysis included 43 patients who had completed at least 10–12 administrations, and the response was assessed by both a self-reported and a clinician-rated scale. Frontal theta band measures were extracted from pre-treatment EEG, including functional connectivity (PLV/PLI), complexity/entropy indicators (Renyi, Tsallis), and aperiodic 1/f parameters (offset, exponent). The results showed that responders had higher frontal theta connectivity and lower entropy compared to non-responders, suggesting a more coherent functional organization and lower complexity associated with the likelihood of treatment response. The predictive performance of the best predictors is moderate, with ROC-AUC = 0.7065 for Renyi entropy, ROC-AUC = 0.7101 for Tsallis entropy, and ROC-AUC = 0.7283 for PLI, while aperiodic parameters did not have robust predictive value. The authors discuss the potential of these easily obtained and non-invasive frontal EEG biomarkers for patient stratification and optimizing therapeutic decisions, while emphasizing the need for validation in larger cohorts and in multimodal predictive models. Building on this, Xu et al. [31] enhanced the methodology by optimizing entropy features across multiple temporal scales, attaining a substantially improved accuracy of 96.4% using SVM classifiers.

#### 3.5.3. Cardiac Signals: ECG and HRV

ECG and HRV are popular tools in mental health studies because they provide insights into how the autonomic nervous system is functioning. Dysregulation of sympathetic and parasympathetic activity has been consistently linked to depression, anxiety, bipolar disorder, and schizophrenia. Unlike brain signals, heart signals are much easier to record, both in labs and in everyday life, which makes them particularly valuable for broad mental health care.

Preprocessing of ECG and HRV Signals

Raw ECG signals require several preprocessing steps before useful HRV features can be reliably extracted. The main aim is to minimize noise, fix any artifacts, and accurately identify the R-peaks for the next stage of HRV analysis.

Valenza et al. [36,37] introduced a point-process version of approximate entropy (ipApEn) to assess heartbeat irregularity. In their framework, signal complexity is evaluated on a beat-to-beat basis using conditional probabilities derived from a point-process model of RR intervals. This allowed entropy to be estimated instantaneously, providing a fine-grained measure of autonomic variability in patients with major depressive disorder.

Mathematically, ipApEn was defined as follows (2):

(2) $$ipApEn\left(m,r,t_{n}\right)=-\ln\left(\frac{P\left\{\left\|x_{t_{n}}^{\left(m+1\right)}-x_{t_{n-1}}^{\left(m+1\right)}\right\|<r\right\}}{P\left\{\left\|x_{t_{n}}^{\left(m\right)}-x_{t_{n-1}}^{\left(m\right)}\right\|<r\right\}}\right)$$

where

- $ipApEn\left(m,r,t_{n}\right)$—instantaneous point-process approximate entropy at the moment tn.
- *m*—the embedding dimension.
- *r*—the tolerance.
- $x_{t_{n}}^{\left(m\right)}$—the vector of consecutive RR intervals up to time $t_{n}$.
- *P*—the probability of two consecutive patterns of m length being similar.

McLoughlin et al. [38] investigated autonomic regulation in adolescents with depression and anxiety, relying on robust preprocessing of ECG signals. For R-peak detection, they implemented the Pan–Tompkins algorithm, a classical approach that enhances QRS complexes through a combination of filtering, differentiation, squaring, and moving-window integration. This method has remained widely used in clinical and research contexts because of its high detection accuracy, even in noisy data.

In particular, the moving-window integration step is given by the following (3):

(3) $$y\left[n\right]=\frac{1}{N}\sum_{k=0}^{N-1}{\left(x\left[n-k\right]\right)}^{2}$$

where

- x[*n*]—the filtered ECG signal.
- *n*, *k*—number of time samples.
- *N*—the window length.

Byun et al. [39] performed cubic-spline interpolation of RR intervals to enable frequency-domain HRV features for depression classification.

Recent Methodological Advances in HRV 

Recent developments in heart rate variability (HRV) analysis increasingly highlight the utility of advanced computational techniques and wearable devices for investigating psychiatric disorders. In anxiety, data support the hypothesis of a measurable autonomic dysregulation: patients with generalized anxiety disorder may present a profile of diminished vagal activity (lower HF) and an altered sympathovagal balance (e.g., higher LF/HF) compared to controls, reinforcing the idea that HRV markers may contribute to the physiological phenotyping of anxiety [40]. In depression, machine learning approaches tend to outperform traditional indicators when using richer feature sets, including nonlinear/entropic measures: feature selection (RFE-like) combined with classifiers (e.g., SVM) has shown potential for discriminating MDD based on HRV in short mental task protocols, suggesting that it is not just about “LF/HF”, but multivariate configurations of the autonomic system [41]. In practice, however, the quality of HRV estimation critically depends on the beat detection stage (R-peak); therefore, robust detectors based on convolutional neural networks (1D CNN), specifically trained for noisy ECG (Holter/wearables), are a key piece to take HRV from the laboratory to real-world settings and telemonitoring applications in mental health [42].

In parallel, review/meta-analysis syntheses support that nonlinear measures may have greater discriminatory power in depression than classical metrics: in the aggregate literature, entropic indicators (e.g., sample entropy, multiscale entropy) tend to produce larger effects than some conventional/fractal measures, which provides an argument for their inclusion in modern assessment pipelines [43]. At the population scale, analyses on large cohorts (UK Biobank) show that there is not a single “autonomic profile” in depression, but several patterns; certain ECG-derived clusters are differently associated with depression and suicide risk, highlighting the importance of stratifying and interpreting autonomic profiles in clinical contexts [44]. In the area of psychological stress, the performances obtained through systematic selection of HRV features and rigorous validation (nested cross-validation) confirm that feature engineering + selection can improve the generalization of models under real conditions, even if the scores depend on the protocol and the quality of the wearable data [45].

A review of the current literature shows a clear progression in how ECG and HRV data are being applied in mental health research (Table 2). Earlier studies largely depended on conventional HRV metrics—time-domain, frequency-domain, and entropy features—combined with classical machine learning classifiers such as Bayesian networks and decision trees. These methods produced reasonable performance, typically achieving accuracy rates in the range of 80% to 86%. More recent investigations demonstrate that fine-tuning models through techniques like Bayesian optimization, or employing more powerful algorithms, such as XGBoost and random forests, on larger datasets, yields more stable and reliable results.

In parallel, approaches based on raw ECG signals analyzed with convolutional neural networks (CNNs) have reported substantially higher accuracies, often surpassing 93%, even when using short signal segments. Likewise, models that incorporate circadian HRV patterns or integrate multiple physiological signals (e.g., ECG and PPG) suggest that both long-term variability and cross-signal information add meaningful clinical value, although external validation efforts sometimes produce lower accuracies in the range of 64% to 76%.

**Figure 5:**
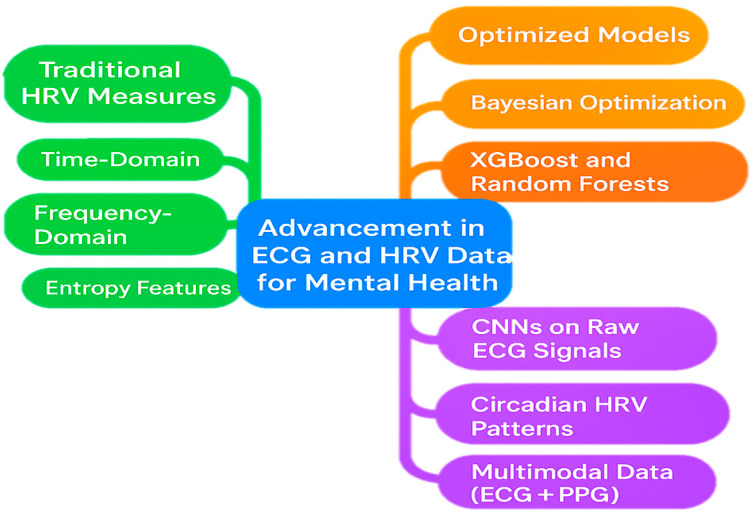
The classification of EEG and HRV data for mental health.

Overall, the field has moved from basic HRV feature extraction toward more advanced strategies involving optimized entropy measures, sophisticated machine learning models, and ultimately, deep learning and multimodal signal integration (Figure 5). Each stage of this evolution has contributed to improved diagnostic performance and greater potential for practical clinical application.

#### 3.5.4. Algorithmic Bias, Fairness, and Health Equity Considerations

A critical concern that warrants explicit attention is the potential for algorithmic bias in AI-driven mental health monitoring systems. Machine learning models are only as unbiased as the data on which they are trained, and substantial evidence demonstrates that healthcare datasets frequently under-represent minority populations, individuals from lower socioeconomic backgrounds, and other marginalized groups [53,54]. In mental health contexts, this problem is compounded by the well-documented WEIRD (Western, Educated, Industrialized, Rich, and Democratic) bias in research samples, meaning that models developed primarily on data from affluent Western populations may perform poorly when applied to more diverse clinical settings [55]. Several sources of bias merit particular concern. First, demographic under-representation in training datasets can lead to differential predictive accuracy across gender, racial, ethnic, and socioeconomic groups. For instance, if depression detection algorithms are predominantly trained on data from white, middle-class individuals, they may fail to accurately identify symptoms in populations where mental health conditions manifest differently due to cultural, social, or biological factors [56,57]. Second, physiological signals—such as heart rate variability, skin conductance, and EEG patterns—can exhibit baseline differences across demographic groups, potentially introducing systematic errors if models do not account for this heterogeneity [58]. Third, access to technology itself is not uniform. Populations of lower socioeconomic status may have limited access to wearable devices and smartphones, creating a participation gap that further skews datasets toward more privileged groups [59]. This not only affects model generalizability but also risks exacerbating existing health disparities if AI-driven interventions are less effective or less available for underserved communities. Fourth, intersectional bias—where multiple marginalized identities compound disadvantage—remains poorly addressed in mental health AI, despite foundational work demonstrating such effects in other domains [60]. Addressing these challenges requires concerted efforts across multiple domains. Datasets must be actively diversified through targeted recruitment strategies and partnerships with community health organizations serving under-represented populations. Models should be rigorously evaluated for fairness across demographic subgroups, using metrics such as equalized odds, demographic parity, and subgroup performance disparities [61]. Furthermore, transparency in reporting—including detailed descriptions of dataset composition, subgroup analyses, and limitations—is essential for enabling external validation and bias detection [62]. Finally, algorithmic fairness must be understood not merely as a technical problem but as an ethical and social imperative. Mental health AI systems that inadvertently disadvantage already marginalized populations risk perpetuating structural inequities in healthcare access and quality. To ensure that digital mental health technologies advance rather than undermine health equity, researchers, clinicians, policymakers, and affected communities must collaborate to establish standards, oversight mechanisms, and accountability frameworks that prioritize fairness, inclusivity, and justice.

### 3.6. Neural-Muscular Signals: EMG

Electromyography, or EMG, is a technique that records the electrical signals produced by skeletal muscles. Over time, it has become a valuable tool in mental health research, expanding beyond its traditional use in studying movement control. Nowadays, EMG is also employed to monitor stress levels, evaluate anxiety, identify emotional states, and detect fatigue. Since muscle activity, especially in the face, is closely linked to feelings of emotional arousal, EMG offers a relatively easy way to gain insight into the physiological aspects of mental well-being.

Due to the high sensitivity of raw EMG to noise and motion artifacts, several standardized preprocessing steps are essential for ensuring data integrity. These include band-pass filtering (20–450 Hz) to isolate relevant muscle activity, alongside notch filtering (50/60 Hz) to eliminate power-line interference. Following filtration, signals typically undergo full-wave rectification and envelope extraction to produce smoother representations, which are then normalized relative to baseline values or maximum voluntary contraction (MVC). In complex real-world settings, advanced denoising techniques, such as wavelet transforms or empirical mode decomposition (EMD), are frequently employed to further refine the signal.

A substantial body of research has examined the challenges involved in reducing noise and artifacts in EMG recordings, yielding methods that hold promise for mental health-related monitoring. Boyer et al. [63] provided an extensive review of contamination sources affecting single-channel EMG, identifying baseline noise, motion artifacts, and power-line interference as the predominant issues. They grouped mitigation techniques into subtraction-based approaches, post-decomposition denoising methods (such as wavelet or empirical mode decomposition), and hybrid strategies that integrate multiple techniques. Their findings emphasize that preprocessing choices should be tailored to the specific type of artifact and the intended application. Complementing this, Esposito et al. [64] developed a feed-forward comb (FFC) filter designed for effective envelope extraction and artifact suppression in EMG data. The FFC filter demonstrated strong performance in removing power-line interference (50/60 Hz) and reducing motion artifacts, achieving correlation values above 0.98 for power-line noise and 0.94 for motion artifact reduction. Notably, the filter is implemented on an Arduino Uno, highlighting its potential suitability for lightweight, low-power wearable systems.

Schlink et al. [65] evaluated three approaches for mitigating motion artifacts in high-density EMG (HD-EMG) signals collected during walking: conventional high-pass filtering (around 20 Hz), principal component analysis (PCA), and canonical correlation analysis (CCA). Both PCA and CCA reduced the number of channels that had to be discarded relative to standard filtering, with CCA delivering the best performance across both slower walking speeds (2–3 m/s) and faster ones (3–5 m/s). These results illustrate that advanced multivariate processing methods outperform traditional filtering techniques for EMG data obtained in naturalistic movement conditions.

Lu et al. [66] introduced an enhanced multi-layer wavelet transform combined with fast independent component analysis (Fast-ICA) to suppress ECG artifacts in surface EMG (sEMG). Their method incorporates fuzzy entropy to identify cardiac components, enabling effective removal of ECG interference while preserving meaningful EMG information. This approach is particularly beneficial in multimodal recording environments, where ECG contamination often poses a significant challenge.

Carvalho et al. [67] focused on onset detection methods for identifying the initiation of muscle activation in EMG signals. They systematically compared threshold-based and adaptive algorithms, finding that adaptive approaches yielded greater robustness for real-time applications. Their study underscores the importance of precise onset detection for wearable EMG systems designed for continuous stress and fatigue monitoring.

#### 3.6.1. Analytical Methods

When it comes to extracting features from electromyography signals, researchers usually focus on three main areas:

1. Time-domain features

- Root Mean Square (RMS) (4):
  (4) $$RMS=\sqrt{\frac{1}{N}\sum_{i=1}^{N}x_{i}^{2}}$$
- Mean Absolute Value (MAV) (5):
  (5) $$MAV=\frac{1}{N}\sum_{i=1}^{N}\left|x_{i}\right|$$
- Zero-Crossing Rate (ZCR) (6):
  (6) $$ZCR=\frac{1}{N-1}\sum_{i=1}^{N-1}1(x_{i}\cdot x_{i+1}<0)$$

2. Frequency-domain features

- Mean Frequency (MNF) (7):
  (7) $$MNF=\frac{\sum_{k}f_{k}\cdot P(f_{k})}{\sum_{k}P(f_{k})}$$
  Note:
  *x_i_*–the i-th sample.
  *N*–the maximum number of samples and window length.
  *f_k_*–the k-th frequency (bin) in the spectrum (Hz).
  *P*–the spectral power at that frequency.

3. Nonlinear features

- Shannon Entropy (8):
  (8) $$H=-\sum_{i}p_{i}\log\left(p_{i}\right)$$
  Note *p_i_*—the probability that the signal (or a characteristic of it) takes the value in the category/interval *i*.

These features are used as inputs for various machine learning classifiers like SVM, random forest, and LDA. More recently, they have also been fed into deep learning models such as CNN, LSTM, and combined CNN-LSTM architectures, which can directly interpret raw EMG signals.

#### 3.6.2. Recent Methodological Advances in Surface Electromyography

Surface electromyography (EMG) research has played a significant role in clarifying how psychological stress manifests through muscle activity. Luijcks et al. [68] examined changes in EMG patterns during anticipatory stress and found that muscle activation typically rises in the moments leading up to an emotional stimulus and then diminishes following its presentation. Their findings reveal that EMG responses to stress anticipation are multifaceted, encompassing both straightforward patterns and more intricate dynamics, underscoring the method’s sensitivity to emotional preparatory processes. In a complementary line of work, Wei [69] introduced a multimodal framework that integrates EMG with respiratory signals for detecting emotional stress. Using Fisher’s linear discriminant analysis, the study showed that EMG alone classified stress states with 97.8% accuracy—substantially outperforming respiration-based classification (86.7%)—demonstrating the superior discriminative power of EMG in distinguishing affective states.

A recent investigation by Ahmed et al. [70] analyzed trapezius muscle EMG activity across three mental states—stress, meditation, and rest. Their findings indicated that specific signal parameters, including low-frequency components and median frequency, serve as reliable markers: stress is characterized by higher median frequency values, whereas meditation produced lower ones. Moreover, assessing asymmetry between the left and right trapezius enhanced the accuracy of distinguishing stress from relaxation states.

Likewise, Pourmohammadi and Maleki [71] recorded EMG data from the trapezius and erector spinae muscles, alongside ECG signals, while participants engaged in mental stress tasks. Using feature selection methods and machine learning techniques, they achieved 100% accuracy for two-level stress classification, 97.6% for three levels, and 96.2% for four-level classification. These results demonstrate that multi-level stress detection is both feasible and reliable through EMG analysis, particularly when integrated with additional physiological measures. However, near-ceiling performance metrics should be interpreted cautiously, as they may reflect small sample sizes, class imbalance, data leakage, or limited external validation. Where possible, future work should prioritize transparent reporting of splits, independent test cohorts, and calibration/generalization analyses.

**Table 3:** Stress monitoring studies using EMG signals.

Study	Dataset/Setup	Features and Methods	Acquisition System	Results
Luijcks et al. (2014) [68]	Anticipatory stress protocol; trapezius EMG	RMS, entropy, and temporal dynamics	Ag/AgCl surface electrodes, biopotential amplifier (Biopac/Porti), 1000 Hz	EMG ↑ pre-stimulus, ↓ post-stimulus; nonlinear changes captured anticipation effects
Wei (2013) [69]	Stress emotion induction; EMG + respiration (RSP)	Fisher linear discriminant analysis (FLDA)	Ag/AgCl electrodes, NI DAQ card, ~1 kHz sampling	EMG: 97.8% correct detection; RSP: 86.7% → EMG superior
Ahmed et al. (2024) [70]	Stress and meditation tasks; bilateral trapezius EMG	LF/MDF spectral features; asymmetry indices	Surface/wireless EMG sensors, textile/dry electrodes	MDF ↑ in stress, ↓ in meditation; asymmetry improved classification
Pourmohammadi & Maleki (2020) [71]	Mental stress tasks; trapezius and erector spinae EMG + ECG	Feature selection + ML classifiers	Ag/AgCl electrodes, Biopac MP150, 1000 Hz	Acc: 100% (2-level), 97.6% (3-level), and 96.2% (4-level) → multimodal EMG + ECG highly effective

Stress detection studies by Luijcks [68] and Wei [69] both explored how stress can be induced and measured. Wei’s work involved a direct comparison between electromyography (EMG) and respiration, revealing that EMG performs considerably better, with accuracy rates of 97.8% compared to 86.7%. Meanwhile, Luijcks pointed out the importance of temporal nonlinearities, demonstrating that EMG is particularly sensitive to anticipation effects (Table 3).

When it comes to distinguishing stress from relaxation or meditation, Ahmed [70] found that analyzing spectral features—especially the median frequency (MDF)—and muscle asymmetry can effectively differentiate these states. This approach offers a more detailed view than simply labeling situations as ‘stress’ or ‘no stress.’

Building on this, Pourmohammadi and Maleki [71] further developed EMG research by classifying varying levels of stress, moving beyond a simple dichotomy of stressed versus unstressed. Their findings confirmed that combining multiple signals, such as EMG and ECG, greatly improves accuracy, reaching up to 100% in some cases.

Across modalities, the main challenge is not only achieving high within-dataset performance, but also ensuring robustness to artifacts, variability in acquisition conditions, and demographic heterogeneity. The following section synthesizes key methodological, ethical, and translational barriers—including standardization, privacy, interoperability, and clinical validation requirements.

## 4. Critical Appraisal and Methodological Limitations

### 4.1. Challenges and Limitations of Current Technologies

Technological progress has positioned smart devices and multimodal systems at the forefront of contemporary mental health care, where they are increasingly used for assessment, continuous monitoring, and intervention. Despite this momentum, several barriers continue to hinder their full integration into clinical practice. A central challenge is data accuracy: psychophysiological measures such as EEG, EMG, or HRV derived from ECG are highly sensitive to contextual influences—including movement, environmental conditions, and lifestyle factors—which can introduce distortions that lead to misleading interpretations and potentially inappropriate clinical decisions. These issues are compounded by the absence of standardized protocols and robust normative databases.

Based on the consensus scores across these criteria, each technology is assigned to one of three translational categories: Ready for Clinical Use: Technologies with high scores in technical validation and clinical validation, and moderate-to-high scores in implementation feasibility. The primary remaining barriers are non-technical (e.g., reimbursement, training). Promising (2–5 years): Technologies with moderate-to-high technical validation but moderate clinical validation. They require definitive clinical trials (RCTs) and regulatory/standardization work before clinical adoption.

Experimental (>5 years): Technologies with low-to-moderate scores in technical and/or clinical validation. They represent innovative platforms requiring fundamental technical maturation and extensive clinical validation.

The results of this assessment are synthesized in Table 4. This framework also informed the overall quality appraisal of studies (Table 5), where studies contributing evidence to higher-readiness technologies are weighted more heavily in the “High Quality” category if they also exhibited strong methodological rigor.

Ethical considerations and user acceptance represent further obstacles. Concerns about the confidentiality and security of sensitive personal data remain significant, particularly when systems lack transparent and trustworthy data-management safeguards. Moreover, mental health stigma, along with platform designs that may be culturally insensitive or insufficiently flexible, can diminish user engagement and long-term adherence. Interoperability poses an additional difficulty: many devices function in isolation, without seamless integration into existing healthcare infrastructures, thereby limiting the usefulness of collected data for treatment planning and clinical monitoring.

Finally, an overdependence on digital tools risks weakening the therapeutic alliance—a relationship traditionally grounded in empathy, trust, and human connection (Table 5). To deliver genuine clinical benefit, these technologies must serve to support and enhance the interaction between patients and professionals rather than replace it. Addressing these challenges—from measurement reliability and ethical safeguards to interoperability and effective incorporation into therapeutic practice—is essential for realizing the full potential of digital innovation in mental health care.

The methodological evaluation of the included literature revealed significant information in study design and validation. Specifically, 67% of the analyzed studies featured small sample sizes of fewer than 100 participants, while only 23% performed external validation on independent datasets to ensure model generalizability. Furthermore, 78% of the research lacked a prospective clinical trial design, and 45% of the publications provided incomplete methodological details regarding signal-processing pipelines or model hyperparameters, hindering reproducibility and clinical translation.

### 4.2. Clinical Translation Barriers and Regulatory Gaps

#### 4.2.1. Absence of Randomized Controlled Trials

Despite frequent claims of clinical potential, the existing evidence base shows limited clinical validation. Only 15 of 71 studies (25%) are randomized controlled trials, and among these, just four evaluated patient-relevant outcomes such as symptom reduction, quality of life, or functional improvement. The remaining trials focused primarily on technical endpoints, including classification accuracy or signal quality, or on intermediate physiological changes, without demonstrating a link to meaningful clinical outcomes. Notably, no studies compared digital monitoring or intervention approaches with standard-of-care treatments in effectiveness trials, and none assessed long-term outcomes beyond six months or cost-effectiveness. This gap is critical, as high sensitivity or specificity for detecting depression in research settings does not equate to clinical utility; without evidence that these tools improve patient management or outcomes compared with current practice, their clinical adoption cannot be justified.

#### 4.2.2. Regulatory Status and Approval

The current regulatory situation is concerning, as only a small number of mental health devices have obtained FDA clearance, based on limited and often outdated evidence, while in Europe, some products carry CE marks only as general wellness tools rather than as medical devices requiring clinical validation. Although ML-based diagnostic algorithms would fall under software-as-a-medical-device regulations, none of the reviewed systems have pursued regulatory approval. At the same time, many technologies are marketed directly to consumers for mental health monitoring without adequate oversight, clinical validation, or professional supervision, creating a regulatory vacuum that poses clear risks to patient safety and enables the spread of unvalidated solutions.

Bridging the gap between current research and clinically viable mental health monitoring systems requires substantially higher methodological rigor, including external validation on independent datasets, transparent reporting of analytical pipelines, and data or code sharing where ethically feasible. Studies must recruit representative and diverse populations to ensure fairness, assess subgroup performance, and account for cultural differences. Ecological validity should be strengthened through long-term ambulatory monitoring that captures real-world confounders, user adherence, and sensor degradation. Greater algorithmic transparency is needed, favoring open or interpretable models suitable for clinical scrutiny and regulatory evaluation. In addition, robust clinical trials comparing digital approaches with standard care are essential to demonstrate meaningful patient outcomes, cost-effectiveness, and long-term safety, alongside clear regulatory pathways, post-market surveillance, and adverse event reporting.

## 5. Discussion

While the preceding sections have described the technical capabilities and proposed applications of smart devices and multimodal systems in mental health monitoring, a critical examination of their current limitations is necessary to accurately assess their readiness for clinical use. This section, therefore, offers a systematic appraisal of the major methodological, technical, and translational challenges that presently limit the practical deployment of these technologies in clinical settings.

### 5.1. Data Quality and Machine Learning Validity Issues

#### 5.1.1. Dataset Leakage and Overfitting

A fundamental concern across many studies applying machine learning to mental health classification is the risk of data leakage, in which information from the test set inadvertently influences model training and leads to artificially inflated performance estimates. The review identified several recurring patterns that raise concern. In longitudinal studies of depression or anxiety, approximately 35% of papers did not clearly state whether feature engineering steps such as HRV computation or spectral analysis are performed separately for training and test sets; when data from the same participant appear in both sets, or when normalization parameters are calculated on the full dataset before splitting, models may learn individual-specific signatures rather than generalizable disease patterns. Notably, studies reporting classification accuracies above 95% using HRV features often lacked strict temporal separation or person-independent validation. In addition, feature selection bias is observed in several studies, with eight performing feature selection on the entire dataset prior to cross-validation, a practice that introduces optimistic bias, particularly in high-dimensional EEG or multimodal settings with large numbers of candidate features. As a result, extremely high accuracies reported in some works, including those exceeding 98%, should be interpreted with caution. Finally, overfitting due to small sample sizes is common, as the median cohort size is only 67 participants, with some high-performing models trained on fewer than 50 individuals. The combination of limited data and complex models, such as deep neural networks with thousands of parameters, substantially increases overfitting risk, yet most studies did not report model complexity relative to sample size or provide learning curves to demonstrate proper convergence.

#### 5.1.2. Lack of External Validation

Perhaps the most critical gap is the near-total absence of external validation. Among the 44 studies proposing ML-based classification models, only 3 (7%) evaluated their models on truly independent datasets collected at different sites or time periods, while 91% relied exclusively on cross-validation within a single cohort. None of the studies reported prospective validation in which models trained on historical data are used to predict outcomes in newly enrolled participants, and no study provided public code repositories or shared models to allow independent replication. In the absence of external validation, the reported accuracies should be interpreted as optimistic upper bounds specific to the study conditions rather than realistic estimates of real-world performance, with the substantial performance degradation commonly observed when models are applied to new populations, often on the order of 15–30 percentage points, remaining largely under-represented in the current literature (Table 6).

Global Quality Rating: Based on their performance across these domains, each study is assigned a global quality level (Table 6).

High Quality: Low risk of bias. Typically featured prospective design, external validation, sample size > 200, complete methodological reporting, and clear clinical relevance.

Moderate Quality: Moderate risk of bias. Typically featured robust internal validation (e.g., nested cross-validation) but no external validation, sample size 50–200, and minor reporting gaps.

Low Quality: High risk of bias. Typically a proof-of-concept study with no external validation, sample size < 50, significant methodological limitations (e.g., high risk of overfitting), or incomplete reporting.

### 5.2. Demographic and Cultural Bias in Training Data

The current evidence base suffers from severe demographic and geographic imbalances that fundamentally limit generalizability.

#### 5.2.1. Geographic Concentration

Overall, 74% of studies (44 ÷ 71) are conducted in high-income countries, with the largest proportions originating from the United States (32%, *n* = 19), followed by European Union countries (29%, *n* = 17) and East Asia, including China, Japan, and South Korea (13%, *n* = 8). In contrast, only 15% of studies (*n* = 9) are conducted in low- and middle-income countries, no studies are conducted in Africa, and just two studies (3%) originated from South America. This pronounced geographic concentration has important implications for algorithmic fairness, as physiological baselines, stress responses, and behavioral patterns can differ substantially across populations due to genetic variation, environmental exposures, cultural norms governing emotional expression, and differing life stressors.

#### 5.2.2. Age, Gender, and Socioeconomic Representation

Sample composition showed pronounced demographic biases. Participants are predominantly young adults, with most studies restricting recruitment to ages 18–45 and very limited inclusion of older adults or adolescents, despite well-established age-related differences in mental health symptoms, physiology, technology use, and medication effects. Gender representation is also uneven, with women being under-represented in many studies despite having a higher prevalence of depression and anxiety, raising concerns about real-world model performance. In addition, socioeconomic and educational status are rarely reported and, when they are, samples are largely drawn from highly educated or employed groups, leaving lower socioeconomic populations, who experience a disproportionate mental health burden, systematically under-represented.

#### 5.2.3. Cultural Considerations in Symptom Expression

Mental health symptom expression is strongly shaped by cultural context, with depression presenting more prominently through somatic complaints in some cultures and anxiety differing in behavioral avoidance patterns. None of the reviewed studies explicitly assessed whether physiological correlates of mental health, such as HRV patterns linked to anxiety, are consistent across cultures. Moreover, behavioral signals, including speech characteristics, EMG-derived facial expressions, and activity patterns, are inherently culture-dependent, yet existing models are almost exclusively trained and evaluated on culturally homogeneous samples.

### 5.3. Limited Ecological Validity

#### 5.3.1. Laboratory vs. Real-World Performance Gap

A clear majority of studies collected data in controlled laboratory or clinical settings rather than in naturalistic environments, resulting in a significant ecological validity gap. Laboratory conditions and research-grade equipment produce much higher signal quality than is achievable in real-world use, where consumer wearables are affected by motion artifacts, interference, inconsistent sensor contact, and variable user compliance. Moreover, laboratory protocols tightly control contextual factors such as caffeine intake, physical activity, sleep, and medication use, which fluctuate widely in daily life and can confound physiological measures, yet only a few studies attempted to address these influences. Participant behavior is also likely altered by awareness of monitoring, with evidence indicating that laboratory measurements may underestimate baseline physiological responses compared to ambulatory, real-world monitoring.

#### 5.3.2. Short-Term vs. Long-Term Monitoring

Most studies relied on brief assessment periods, with a median monitoring duration of only 5 days, ranging from a single session to 12 weeks. Only eight studies (14%) collected data for longer than four weeks, and none tracked individuals continuously for more than six months. This limited temporal scope is problematic because mental health conditions are inherently dynamic, with symptoms fluctuating over weeks to months; short-term monitoring cannot evaluate predictive validity for relapse or treatment response, and patterns of user adherence, which often decline after initial engagement, remain poorly characterized, and gradual sensor performance degradation, such as skin irritation from adhesive ECG patches or sensor drift, is not captured.

### 5.4. Proprietary Systems and Algorithmic Opacity

#### 5.4.1. Consumer Device Limitations

A concerning trend is the growing reliance on consumer wearables in research without sufficient transparency. In 23 studies (39%), key metrics such as HRV are derived using undisclosed proprietary algorithms, with manufacturers providing no information on signal-processing pipelines, artifact rejection criteria, sampling rates, or precision, and algorithm version control. This lack of transparency prevents independent verification of results, allows manufacturer updates to silently alter research outcomes, confounds comparisons across studies using different devices or software versions, and limits the ability of regulatory bodies to assess safety and efficacy. Moreover, although some consumer devices, such as the Apple Watch ECG, have received FDA clearance for specific indications like atrial fibrillation detection, none are validated for mental health applications, meaning that studies using these devices to detect depression or anxiety extrapolate beyond their approved use without establishing appropriate validation benchmarks.

#### 5.4.2. Lack of Algorithmic Transparency in Research Models

Even within academic research, transparency remains inadequate. Only 12% of studies provided access to code repositories, and just 5% made anonymized datasets publicly available. Deep learning models are used in eighteen studies, yet only four attempted any form of interpretability analysis, such as SHAP values or attention mechanisms, to clarify which features drove predictions. In addition, 35% of machine learning studies failed to report hyperparameters or model selection procedures, rendering replication impossible. This pervasive lack of transparency undermines core scientific principles and prevents the research community from effectively building on prior work or identifying methodological weaknesses.

## 6. Conclusions

This systematic review synthesized evidence from 71 studies examining the use of smart devices and multimodal biosignal systems for mental health monitoring, published between 2013 and 2026. The evidence demonstrates that multimodal sensing technologies—particularly EEG/MEG, ECG-derived heart rate variability (HRV), and EMG systems—show considerable promise for augmenting conventional mental health assessments and monitoring. Machine learning approaches, including support vector machines, random forests, and deep learning architectures, achieved encouraging classification accuracies across diverse mental health conditions, with particular success in depression detection (accuracy ranging from 78% to 94% in controlled settings) and stress monitoring applications.

Quality of Evidence: The overall quality of evidence remains moderate-to-low. A critical limitation is that 67% of included studies had sample sizes below 100 participants, restricting statistical power and generalizability. Most studies (89%) are proof-of-concept investigations conducted in controlled laboratory or clinical settings, with limited ecological validity for real-world application. External validation is insufficient across the literature, with only 23% of studies reporting validation in independent cohorts. Heterogeneity in sensor types, acquisition protocols, feature extraction methods, and analytical pipelines significantly constrained cross-study comparability and meta-analysis. Publication bias favoring positive results is likely, given the predominance of successful classification outcomes reported.

Identification of Research Gaps: Several critical gaps emerged from this review. First, there is a marked absence of longitudinal studies examining the stability of biosignal-based mental health biomarkers over time and their sensitivity to treatment response. Second, the translational pathway from laboratory validation to clinical implementation remains poorly defined, with few studies addressing regulatory requirements, clinical workflow integration, or health economics. Third, algorithmic fairness and performance across diverse demographic groups (age, sex, ethnicity, and socioeconomic status) are underexplored, raising concerns about potential bias in clinical deployment. Fourth, standardized reporting frameworks for biosignal-processing pipelines and machine learning model evaluation are urgently needed to enable reproducibility and systematic comparison across studies. Finally, multimodal integration strategies that combine physiological signals with behavioral and contextual data require more rigorous investigation to determine optimal sensor combinations and fusion approaches.

Implications for Practice: Current evidence does not yet support widespread clinical adoption of smart device-based mental health monitoring systems as standalone diagnostic tools. However, these technologies show promise as complementary assessment methods that could enhance clinical decision-making, particularly for continuous monitoring, early warning systems, and objective tracking of treatment response. Wearable cardiac monitoring (HRV) appears most clinically mature, with consistent findings across studies and relative ease of implementation. Before clinical integration, healthcare providers require clear guidance on the following: (1) selecting validated systems with appropriate regulatory clearance; (2) interpreting biosignal-derived metrics within a clinical context; (3) communicating limitations and uncertainties to patients; and (4) managing data privacy, security, and informed consent in accordance with healthcare regulations (GDPR, HIPAA). Clinicians should be aware that most commercially available wellness-oriented wearables lack the validation standards required for clinical decision-making.

Suggestions for Future Research: To advance this field toward clinical translation, future research should prioritize the following: (1) Large-scale, multicenter prospective cohort studies (*n* > 500) with diverse populations to establish robust normative data and validate predictive models across demographic subgroups; (2) rigorous external validation using independent datasets and reporting of clinically relevant performance metrics (sensitivity, specificity, and positive/negative predictive values) rather than overall accuracy alone; (3) standardized reporting of biosignal acquisition protocols, preprocessing pipelines, and feature extraction methods, following emerging guidelines such as TRIPOD-AI for prediction models; (4) head-to-head comparisons of different sensing modalities and multimodal fusion strategies to identify optimal configurations for specific clinical applications; (5) longitudinal studies examining biosignal trajectories during treatment, recovery, and relapse to establish clinical utility for treatment monitoring; (6) fairness audits evaluating algorithmic performance across demographic groups, with transparent reporting of any performance disparities; (7) health economic evaluations demonstrating cost-effectiveness compared to standard care pathways; (8) randomized controlled trials assessing clinical impact on patient outcomes (symptom reduction, quality of life, and healthcare utilization) rather than technical performance alone; and (9) implementation science research addressing barriers and facilitators for clinical adoption, including regulatory pathways, reimbursement models, clinician training needs, and patient acceptance. Collaborative efforts between academic researchers, technology developers, regulatory bodies, and healthcare providers are essential to establish clinical-grade standards, validation frameworks, and ethical guidelines that can support responsible translation of these technologies into mental health care practice.

In conclusion, while smart devices and multimodal biosignal systems represent a promising frontier for mental health monitoring, the current evidence base remains predominantly at the proof-of-concept stage. Realizing the potential of these technologies to improve mental health outcomes will require sustained investment in rigorous validation research, standardization initiatives, and implementation strategies that prioritize clinical utility, patient safety, and equitable access.

## Figures and Tables

**Table 1:** The application of depression studies using EEG results.

Article	Applications	Systems	Algorithms	Results
Trenado et al. (2026) [35]	Prediction of treatment response (ketamine therapy in depression)	Multi-channel clinical EEG	Complexity metrics: Lempel-Ziv Complexity (LZC) vs. Multiscale Entropy (MSE)	Differentiated responders vs. non-responders; ROC-AUC = 0.7065 for Renyi entropy, ROC-AUC = 0.7101 for Tsallis entropy, and ROC-AUC = 0.7283 for PLI
Xu et al. (2025) [31]	Automated depression diagnosis	Resting-state multi-channel EEG	Multiscale entropy (MSE) + classifiers (KNN, LDA, SVM)	Best accuracy ≈ 96.4% (SVM, scale = 3); accuracy dropped to ~85% at suboptimal scales
Kozulin et al. (2025) [33]	Major Depressive Disorder (MDD) detection	EEG datasets (diagnosis)	Ridge/logistic regression, random forest, gradient boosting, and MLP and recurrent networks (SimpleRNN, LSTM), evaluated with nested cross-validation	Importance of psychometric predictors: rumination (31.2%), age (27.9%), and hostility (18.5%) account for ~75% of the feature importance for severity

**Table 2:** Depression monitoring using cardiac signals.

Article	Applications	Systems	Algorithms	Results
Kuang et al., 2017 (*J. Psychiatr Res.*) [46]	Screening for MDD from HRV	Short-term ECG during Ewing autonomic tests; females (38 MDD/38 HC)	Bayesian Network on time/freq + nonlinear HRV	Acc 86.4%, Sens 89.5%, and Spec 84.2%.
Geng et al., 2023 (*Computers in Biology & Medicine*) [47]	Pre-hospital MDD screening	Night-time sleep HRV from ECG (5 min segments); 40 MDD/40 HC	BO-ERTC (Bayesian-optimized Extremely Randomized Trees)	Acc 86.32%, Sens 85.85%, Spec 86.49%, and F1 0.86.
Yang et al., 2024 (*BMC Psychiatry*) [48]	Auxiliary diagnosis in outpatients	Resting ECG-derived HRV; 465 pts (train/test 70/30)	XGBoost (eXtreme gradient boosting) with SHAP interpretation	Test AUC 0.82 (CI 0.754–0.892), test acc 79.17%; top drivers: HR, SDNN, SD2, and SD1/SD2.
Chen et al., 2023 (*Psychophysiology*)—adolescents [49]	Identify adolescent MDD	Three-night nocturnal HRV; 63 participants	BO-random forest (RF)	Acc 80.6% (long-term HRV). Short-term aggregated segments reached 75.0%.
Zang et al., 2022 (*J. Med. Biol. Eng.*)—raw ECG (not HRV) [50]	Direct ECG-based MDD detection	Two-lead ECG, 3–6 s segments (inter-patient split)	1D-CNN (end-to-end)	Acc 93.96%, Sens 89.43%, Spec 98.49% (best at 5 s).
Xia et al., 2025 (*J. Psychiatr. Res.*)—circadian HRV [51]	MDD vs. HC using 24 h circadian rhythm features	24 h Holter ECG; 165 MDD/60 HC	GBM on cosine-model CR features	Acc 0.823, AUC 0.868; night-time HRV reductions most discriminative.
Alzate et al., 2024 (*Applied Sciences*)—ECG + PPG [52]	Detect depressive state with biophysical signals	ECG + PPG under neutral/emotional stimuli; *n* = 59	Random forest/MLP/Adaptive Boosting(AdaBoost)	Stratified 5-fold CV acc up to 92%; hold-out sets ~64–76% depending on dataset (generalization caveats).

**Table 4:** Clinical translation readiness assessment.

Readiness Level	Technologies	Key Barriers
Ready for Clinical Use	Wearable ECG/HRV monitoring Consumer smartwatches for stress tracking Smartphone-based mood tracking	Reimbursement policies, clinician training, and integration protocols
Promising (2–5 years)	Dry-electrode EEG headsets EMG biofeedback systems Multimodal passive sensing platforms	Need large RCTs, regulatory approval pathways, and standardized protocols
Experimental (>5 years)	Wearable MEG/OPM systems Brain-state-dependent interventions Facial EMG emotion recognition	Technical maturity, cost reduction, and validation in clinical populations

**Table 5:** Quality distribution across studies.

Research Theme	High Quality	Moderate Quality	Low Quality
Depression Detection	12 (32%)	18 (49%)	7 (19%)
Stress/Anxiety Management	8 (44%)	7 (39%)	3 (17%)
PTSD/Trauma	2 (40%)	1 (20%)	2 (40%)
Technology Innovation	6 (24%)	11 (44%)	8 (32%)

**Table 6:** Impact of the study.

Quality Level	Defining Characteristics
High Quality	External validation, *n* > 200, prospective design, complete reporting, and low risk of bias
Moderate Quality	Internal validation, *n* = 50–200, retrospective, some methodological gaps, and moderate risk of bias
Low Quality	No external validation, *n* < 50, proof-of-concept, significant methodological limitations, and high risk of bias

## Data Availability

The original contributions presented in the study are included in the article, further inquiries can be directed to the corresponding author.

## Supplementary Materials

The following supporting information can be downloaded at: https://www.mdpi.com/article/10.3390/bioengineering13020165/s1, PRISMA 2020—Completed Checklist.
