# Supplementary material for: Smart Devices and Multimodal Systems for Mental Health Monitoring: From Theory to Application
Source: Bioengineering (Basel). 2026 Jan 29;13(2):165. doi: 10.3390/bioengineering13020165 (PMC12938191; doi:10.3390/bioengineering13020165)
Supplement: Supplementary file 1 [file bioengineering-13-00165-s001.zip › bioengineering-4050286-supplementary.pdf]

PRISMA 2020 - Completed Checklist

Section and Topic	Item #	Checklist Item	Location in Manuscript (e.g., Section, Page, Line)
**TITLE**			
Title	1	Identify the report as a systematic review.	Title Page/Abstract ("Smart Devices and Multimodal Systems for Mental Health Monitoring: From Theory to Application")
**ABSTRACT**			
Abstract	2	See the PRISMA 2020 for Abstracts checklist.	Abstract (Structured summary provided)
**INTRODUCTION**			
Rationale	3	Describe the rationale for the review in the context of existing knowledge.	Section 1: Introduction, Paragraphs 6-8 (lines discussing variability, challenges, and prior reviews)
Objectives	4	Provide an explicit statement of the objective(s) or question(s) the review addresses.	Section 1: Introduction, Final paragraph ("In response to the rapid proliferation... this review addresses three primary research questions: (RQ1)... (RQ2)... (RQ3)...")
**METHODS**			
Eligibility criteria	5	Specify the inclusion and exclusion criteria for the review and how studies were grouped for the syntheses.	Section 2: Materials and methods, Paragraphs 2-3 ("The inclusion criteria... Exclusion criteria...")
Information sources	6	Specify all databases, registers, websites, organisations, reference lists and other sources searched or consulted to identify studies. Specify the date when each source was last searched or consulted.	Section 2: Materials and methods, Paragraph 1 ("PubMed/MEDLINE, IEEE Xplore Digital Library, Web of Science, Scopus, PsycINFO and ACM Digital Library databases are searched from 2013-2026")
Search strategy	7	Present the full search strategies for all databases, registers and websites, including any filters and limits used.	Section 2: Materials and methods, Search strategy subsection with complete Boolean operators ("Primary Search String... Database-Specific Adaptations")
Selection process	8	Specify the methods used to decide whether a study met the inclusion criteria of the review, including how many reviewers screened each record and each report	Section 2: Materials and methods, Figure 2 caption notes and text describing PRISMA flow

		retrieved, whether they worked independently, and if applicable, details of automation tools used in the process.	
Data collection process	9	Specify the methods used to collect data from reports, including how many reviewers collected data from each report, whether they worked independently, any processes for obtaining or confirming data from study investigators, and if applicable, details of automation tools used in the process.	Section 2: Materials and methods, Data Extraction Method subsection ("Standardized data extraction form created in Microsoft Excel... Independent extraction by two reviewers; weekly calibration meetings...")
Data items	10a	List and define all outcomes for which data were sought. Specify whether all results that were compatible with each outcome domain in each study were sought (e.g. for all measures, time points, analyses), and if not, the methods used to decide which results to collect.	Section 2: Materials and methods, Outcomes subsection ("Primary outcome measures... Classification accuracy... Sensitivity and specificity... AUC-ROC values...")
	10b	List and define all other variables for which data were sought (e.g., participant and intervention characteristics, funding sources). Describe any assumptions made about any missing or unclear information.	Section 2: Materials and methods, Data Items Extracted subsection with comprehensive list (Study Characteristics, Population Characteristics, Technology Details, Machine Learning Methods, Outcomes, Bias and Quality)
Study risk of bias assessment	11	Specify the methods used to assess risk of bias in the included studies, including details of the tool(s) used, how many reviewers assessed each study and whether they worked independently, and if applicable, details of automation tools used in the process.	Section 5: Discussions, Subsections 5.1 and 5.2 (Data Quality and Machine Learning Validity Issues, Demographic and Cultural Bias) and Table 6 (Quality Level assessment)
Effect measures	12	Specify for each outcome the effect measure(s) (e.g., risk ratio, mean difference) used in the synthesis or presentation of results.	Section 3: Overview of Smart Devices, Tables 1-6 presenting accuracy, sensitivity, specificity, AUC metrics for individual studies
Synthesis methods	13a	Describe the processes used to decide which studies were eligible for each synthesis (e.g., tabulating the study intervention characteristics and comparing against the planned groups for each synthesis (item #5)).	Section 2: Materials and methods ("A thematic analysis of the included literature revealed six predominant cross-cutting themes: Depression detection... 37%; Stress/anxiety... 18%; PTSD... 5%...")
	13b	Describe any methods required to prepare the data for presentation or synthesis, such	Section 2: Materials and methods, paragraph noting descriptive

		as handling of missing summary statistics, or data conversions.	summarization approach (no meta-analysis due to heterogeneity)
	13c	Describe any methods used to tabulate or visually display results of individual studies and syntheses.	Section 3: Overview of Smart Devices, Tables 1-6 organized by modality (EEG/MEG, ECG/HRV, EMG) and clinical application
	13d	Describe any methods used to synthesize results and provide a rationale for the choice(s). If meta-analysis was performed, describe the model(s), method(s) to identify the presence and extent of statistical heterogeneity, and software package(s) used.	Section 2: Materials and methods ("The review integrates findings across modalities... through qualitative description and comparison, rather than statistical pooling") and explanation of why meta-analysis was not conducted
	13e	Describe any methods used to explore possible causes of heterogeneity among study results (e.g., subgroup analysis, meta-regression).	Section 2: Materials and methods, Specific Heterogeneity Issues Identified subsection (1. Diverse Outcome Measures, 2. Varied Methodologies, 3. Study Design Differences, 4. Technical Variability)
	13f	Describe any sensitivity analyses conducted to assess robustness of the synthesized results.	Not conducted. Narrative synthesis approach was used due to heterogeneity precluding meta-analysis.
Reporting bias assessment	14	Describe any methods used to assess risk of bias due to missing results in a synthesis (arising from reporting biases).	Section 6: Conclusion, Quality of Evidence subsection ("Publication bias favoring positive results is likely, given the predominance of successful classification outcomes reported")
Certainty assessment	15	Describe any methods used to assess certainty (or confidence) in the body of evidence for an outcome.	Section 6: Conclusion, Quality of Evidence subsection ("The overall quality of evidence remains moderate to low") and Table 6 with quality level definitions
**RESULTS**			
Study selection	16a	Describe the results of the search and selection process, from the number of records identified in the search to the number of studies included in the review, ideally using a flow diagram.	Section 2: Materials and methods, Figure 2: PRISMA 2020 flow diagram showing complete study selection process with note on study characteristics by sensor type, condition, ML method, and publication year

	16b	Cite studies that might appear to meet the inclusion criteria, but which were excluded, and explain why they were excluded.	Not explicitly provided in main text. Exclusion reasons described in methods; detailed list could be in supplementary material.
Study characteristics	17	Cite each included study and present its characteristics (e.g., study design, population, interventions, outcomes, funding sources).	Section 3: Overview of Smart Devices, comprehensive synthesis organized by sensor modality and clinical application with Tables 1-6
Risk of bias in studies	18	Present assessments of risk of bias for each included study.	Section 5: Discussions, comprehensive critical appraisal in subsections 5.1-5.4 (Data Quality, Demographic Bias, Ecological Validity, Algorithmic Opacity) with Table 6 quality assessment
Results of individual studies	19	For all outcomes, present, for each study: (a) summary statistics for each group (where appropriate) and (b) an effect estimate and its precision (e.g., confidence/credible interval), ideally using structured tables or plots.	Section 3: Overview of Smart Devices, Tables 1-6 presenting performance metrics (accuracy, sensitivity, specificity, AUC) for individual studies
Results of syntheses	20a	For each synthesis, briefly summarize the characteristics and risk of bias among contributing studies.	Section 3: Overview of Smart Devices (thematic synthesis by modality) and Section 5: Discussions (critical appraisal of methodology and bias)
	20b	Present results of all statistical syntheses conducted. If meta-analysis was done, present for each the summary estimate and its precision (e.g., confidence/credible interval) and measures of statistical heterogeneity. If comparing groups, describe the direction of the effect.	No statistical meta-analysis conducted. Section 2 explains heterogeneity precluding pooled analysis. Results presented as narrative synthesis with descriptive statistics.
	20c	Present results of all investigations of possible causes of heterogeneity among study results.	Section 5: Discussions, subsections 5.1-5.4 comprehensively examining sources of heterogeneity (methodological variability, geographic concentration, ecological validity gaps, proprietary systems)
	20d	Present results of all sensitivity analyses conducted to assess the robustness of the synthesized results.	Not conducted. Narrative synthesis approach used.

Reporting biases	21	Present assessments of risk of bias due to missing results (arising from reporting biases) for each synthesis assessed.	Section 6: Conclusion, Quality of Evidence subsection ("Publication bias favoring positive results is likely, given the predominance of successful classification outcomes reported")
Certainty of evidence	22	Present assessments of certainty (or confidence) in the body of evidence for each outcome assessed.	Section 6: Conclusion, Quality of Evidence and Identification of Research Gaps subsections with comprehensive assessment
**DISCUSSION**			
Discussion	23a	Provide a general interpretation of the results in the context of other evidence.	Section 6: Conclusion, comprehensive interpretation across subsections (Quality of Evidence, Research Gaps, Implications for Practice, Suggestions for Future Research)
	23b	Discuss any limitations of the evidence included in the review.	Section 5: Discussions, comprehensive critical appraisal subsections 5.1-5.4 and Section 6: Conclusion, Identification of Research Gaps
	23c	Discuss any limitations of the review processes used.	Section 2: Materials and methods notes that protocol was not prospectively registered but methodology was defined a priori. Further discussion of review limitations could be strengthened.
	23d	Discuss implications of the results for practice, policy, and future research.	Section 6: Conclusion, dedicated subsections on Implications for Practice and Suggestions for Future Research with detailed recommendations
**OTHER INFORMATION**			
Registration and protocol	24a	Provide registration information for the review, including register name and registration number, or state that the review was not registered.	Section 2: Materials and methods explicitly states: "The protocol for this review was not prospectively registered in a public repository. However, the review methodology was defined a priori..."
	24b	Indicate where the review protocol can be accessed, or state that a protocol was not prepared.	Protocol not publicly deposited. Methodology defined a priori as stated in Section 2.

	24c	Describe and explain any amendments to information provided at registration or in the protocol.	Not applicable. No prospective registration.
Support	25	Describe sources of financial or non-financial support for the review, and the role of the funders or sponsors in the review.	Not provided in current version of manuscript.
Competing interests	26	Declare any competing interests of review authors.	Not provided in current version of manuscript.
Availability of data, code and other materials	27	Report which of the following are publicly available and where they can be found: template data collection forms; data extracted from included studies; data used for all analyses; analytic code; any other materials used in the review.	Not provided in current version of manuscript.
